# Gaussian curvature and the budding kinetics of enveloped viruses

**DOI:** 10.1371/journal.pcbi.1006602

**Published:** 2019-08-21

**Authors:** Sanjay Dharmavaram, Selene Baochen She, Guillermo Lázaro, Michael Francis Hagan, Robijn Bruinsma

**Affiliations:** 1 Department of Mathematics, Bucknell University, Lewisburg, Pennsylvania, United States of America; 2 Department of Physics and Astronomy, University of California, Los Angeles, Los Angeles, California, United States of America; 3 Martin A. Fisher School of Physics, Brandeis University, Waltham, Massachusetts, United States of America; 4 Department of Chemistry and Biochemistry, University of California, Los Angeles, Los Angeles, California, United States of America; Hebrew University of Jerusalem, ISRAEL

## Abstract

The formation of a membrane-enveloped virus starts with the assembly of a curved layer of capsid proteins lining the interior of the plasma membrane (PM) of the host cell. This layer develops into a spherical shell (capsid) enveloped by a lipid-rich membrane. In many cases, the budding process *stalls* prior to the release of the virus. Recently, Brownian dynamics simulations of a coarse-grained model system reproduced protracted pausing and stalling, which suggests that the origin of pausing/stalling is to be found in the physics of the budding process. Here, we propose that the pausing/stalling observed in the simulations can be understood as a *purely kinetic* phenomenon associated with the neck geometry. A *geometrical* potential energy barrier develops during the budding that must be overcome by capsid proteins diffusing along the membrane prior to incorporation into the capsid. The barrier is generated by a conflict between the positive Gauss curvature of the assembling capsid and the negative Gauss curvature of the neck region. A continuum theory description is proposed and is compared with the Brownian simulations of the budding of enveloped viruses.

## Author summary

Despite intense study, the life-cycle of the HIV-1 virus continues to pose mysteries. One of these is the fact that the assembly of an HIV-1 virus along the plasma membrane (PM) of the host cell—the budding process—stalls prior to release of the virus. Many other important viral pathogens with a surrounding lipid membrane envelope display similar stalling. Combining numerical and analytical methods, we demonstrate that the neck-like shape of the membrane that forms prior to release of the virus creates a barrier that blocks the proteins required for the assembly process from reaching the budding virus. An improved understanding of the physics of the blocking process could enable new strategies to combat enveloped viruses.

## Introduction

**Fig 1:**
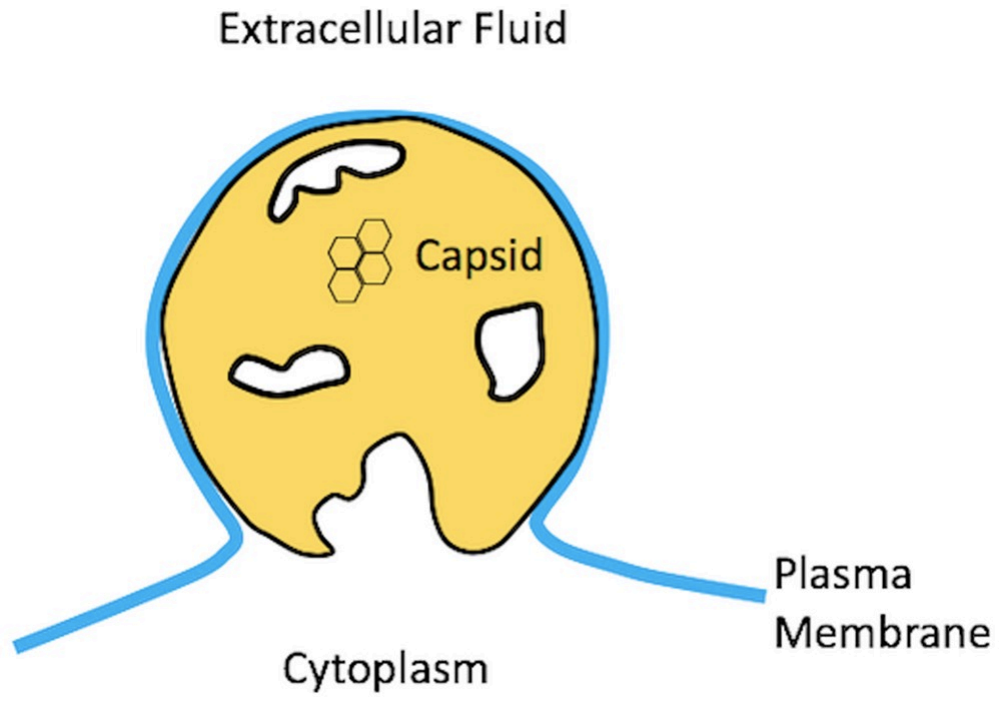
Sketch of a budding HIV-1 virion just prior to pinch-off. The lipid bilayer covering the capsid is connected to the plasma membrane by a highly curved neck. The large hole surrounding the pinch-off site is also a feature of the completed virion.

Many viruses that infect animals, including many human pathogens, are surrounded by a lipid membrane. This membrane allows the viral genome molecules to enter a host cell by membrane fusion [2] and it also prevents attack by the host immune system. Well-known examples are the retroviruses, like HIV-1, the Herpesviruses, and the Filoviruses (e.g. Ebola virus). The enveloping membrane forms during the budding process when a curved layer of viral proteins (“capsid proteins”) line the interior of the PM of the host cell [3–5]. For single-stranded RNA viruses (like HIV-1), the assembly is initiated by viral RNA genome molecules associating with capsid proteins [6]. The capsid grows by transport of proteins from the cytosol, where capsid proteins are being synthesized, to the growing protein shell. The capsid proteins evolve into a spherical-cap shape covered by a membrane that is connected to the PM via a curved neck. Fig 1 shows a sketch of the image of a late-stage bud of an HIV-1 viral particle (“virion”) as obtained by cryo-EM tomography [3]. Up to this point, the budding is a spontaneous process driven by attractive interactions between the capsid proteins with each other and with the viral RNA molecules. For many—but not all—enveloped viruses the final scission of the membrane neck is not a spontaneous process but involves recuitment of the *ESCRT* machinery of the cell [7, 8]. ESCRT is a complex of proteins that plays a role in cellular processes that require membrane scission, such as the formation of multi-vesicular bodies and cytokinesis [9]. Scission of HIV-1 buds does take place in the absence of the ESCRT machinery but with a delay [10, 11]. This suggests that ESCRT recruitment is necessary to assure that scission takes place *on time*. For the case of HIV-1, scission of the neck has to take place before inititation of a spontaneous autocatalytic protease maturation process that breaks up the capsid polyproteins [12]. Moreover, the existence of a large hole at the pinch-off site (Fig 1) suggests that the ESCRT machinery does not continue the shell assembly process, but instead enables scission before the assembly process completes.

The fact that ESCRT recruitment takes place across so many different families of enveloped viruses suggests that the origin of the pausing/halting kinetics must be found among basic properties of the budding process shared among different families of enveloped viruses. In this paper we propose a physical mechanism as the cause for the pausing/stalling.

## Results and discussion

Fig 1, showing a sketch of an HIV-1 bud, provides clues. The lattice of capsid proteins of an HIV-1 bud has a large hole surrounding the pinch-off site. The boundary of the hole represents the growth interface separating the part of the PM that is covered by capsid proteins and the part that is not. Because of hole formation, only about 2/3 of the membrane of the completed immature HIV-1 virus is covered by proteins. Hole formation is observed also for a number of other enveloped viruses, though not for all. Fig 1 suggests that the transport current supplying proteins to the growth interface somehow has “dried-up” before the spontaneous part of the assembly could complete. While the neck of the membrane is too large for spontaneous scission to occur in such a configuration, the ESCRT machinery could drive scission at that point, resulting in a budded virion with a large hole in its shell.

**Fig 2:**
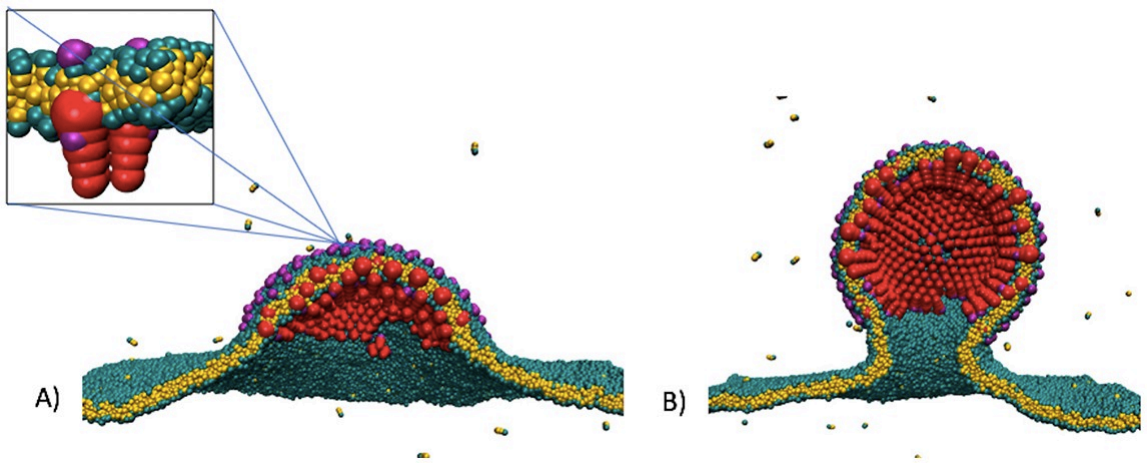
Brownian Dynamics simulation of the budding of an enveloped virus using a coarse-grained model of the lipid molecules and capsid proteins of the alpha virus (square inset). The strength *ϵ*_gg_ of the interaction between the capsid proteins was 2*k*_*B*_ *T*. A) Snapshot of the simulation at an early stage of the bud. B) Snapshot of the bud during pausing.

This notion is supported by numerical simulations. Fig 2 shows snapshots of a Brownian Dynamics simulation of a simple, coarse-grained model of the budding of the enveloped protein shell of the alphavirus, which is composed of transmembrane glycoproteins (GPs) [1]. The GPs were modeled as rigid trimers of truncated cones, with each cone comprising a linear array of six beads of increasing diameter. The cone angle was set so that in the absence of a membrane, the GPs assembled into hollow, roughly icosahedral shells containing 80 trimers, though they form larger shells in the presence of a membrane [1]. The membrane was represented by the implicit solvent model of Cooke and Deserno [13]. As the assembly proceeded, the aggregate of GPs adopted the shape of a *spherical cap* that gradually closed. For high values of the protein-protein binding energy *ϵ*_gg_, complete closure was achieved and pinch-off was spontaneous. The resulting spherical shells were highly defected. The growth rate was non-uniform: the assembly rate started to slow down when the shells reached approximately 2/3 completion. Slow-down became more pronounced with decreasing *ϵ*_gg_ while the final spherical shells were less defected. A critical value was reached for *ϵ*_gg_ about 1.7*k*_B_*T*. Below this value, the assembly process stalled before closure could be achieved. The diameter of the remaining neck grew larger as *ϵ*_gg_ was further decreased.

Figs 1 and 2 suggest a physical mechanism for the stalling: the part of the membrane linking the bud to the PM has a very different geometry from the part of the membrane covering the proteins shell: the latter has a spherical curvature, while the neck was a highly curved hyperbolic shape. Could the curved neck region be energetically costly, delaying or preventing scission? From the viewpoint of the physics of membranes, this seems less likely. Simple lipid bliayer vesicles develop freely from multi-component lipid bilayers through the formation of a neck. The stalling that characterizes the formation of enveloped viruses has not been reported for vesicle budding. In the following, we extend the continuum theory of vesicle formation from multi-component lipid bilayers [14–18] to the case of enveloped viruses to see whether or not there is an energy barrier that inhibits scission.

### Continuum theory: Mechanical equilibrium

The continuum theory of the budding of vesicles from multi-component lipid bilayers [14–18] is based on the Helfrich bending energy. The latter has been used extensively to describe lipid bilayers and deformable surfaces in general [19]. It has been applied to the budding of viruses [20–22] and to the formation of clathrin cages [23]. For a recent review of the application of the bending energy concept to biomembranes, see ref. [24]. Within Helfrich theory, the bending free energy *F*_*B*_ of a deformable, inhomogenerous surface is expressed as

$$\begin{array}{c} F_{B}=\int_{A}\left[2\kappa \left(s\right){\left(H-\frac{1}{R_{0}\left(s\right)}\right)}^{2}+\bar{\kappa }\left(s\right)K\right]dA. \end{array}$$ (1)

The first term describes the energy cost for deviations of the mean curvature $H=\left(1/2\right)\left(\frac{1}{R_{1}}+\frac{1}{R_{2}}\right)$ of a surface from the mean curvature 1/*R*_0_(**s**) that corresponds to the free energy minimum of the local molecular structure. Here, *R*_1,2_ are the principle curvature radii at a given point **s** on the surface of the disk. For a bare lipid bilayer, 1/*R*_0_(**s**) is zero while for the membrane-covered protein layer of a budding spherical virus, 1/*R*_0_(**s**) corresponds to the inverse of the radius of the capsid. The coefficient *κ*(**s**), the local bending modulus, is always positive. For lipid bilayers, the bending modulus *κ*_*L*_ has been measured to be of the order of 20 *k*_*B*_*T*. Values for the bending modulus *κ*_*C*_ for a layer of capsid proteins are not as well established but micromechanical studies indicate that it is significantly larger than that of a lipid bilayer.

In the second term of the bending free energy, *K* = 1/(*R*_1_ *R*_2_) is the Gauss curvature. *K* is positive for a spherical surface and negative for a hyperbolic or saddle-shaped surface. The membrane of the neck region of a budding virus has a negative Gauss curvature. According to the *Gauss-Bonnet Theorem* (GBT), the integral of the Gaussian curvature *K* over a surface A with boundary S obeys $\int_{A}KdA\;+\;\int_{S}k_{g}ds\;=\;2\pi \chi$ with *κ*_*g*_ the *geodesic curvature* of the boundary and with *χ* a topological invariant (know as the “Euler characteristic”). For a closed membrane without a boundary that has a uniform Gauss curvature modulus, the area integral of the Gauss curvature is an invariant equal to 4*π*. As a result, if a spherical bud with positive Gauss curvature is developing along a uniform membrane, then that has to be “paid for” by a section of negative Gauss curvature, such as the neck. If the Gauss modulus is not a constant, as in the present case, then the second term of the GBT is non-zero. Consider a closed membrane composed of two different parts that are joined along a closed boundary line *S*. One part has Gauss modulus $\bar{\kappa }\left(s\right)={\bar{\kappa }}_{C}$ while the other part has Gauss modulus $\bar{\kappa }\left(s\right)={\bar{\kappa }}_{L}$. Application of the GBT leads to

$$\begin{array}{c} \int_{A}\bar{\kappa }\left(s\right)KdA=\left[{\bar{\kappa }}_{C}+{\bar{\kappa }}_{L}\right]2\pi -\left[{\bar{\kappa }}_{C}-{\bar{\kappa }}_{L}\right]\int_{S}\kappa_{g}ds. \end{array}$$ (2)

For such a non-uniform surface, the Gaussian curvature energy $\int_{A}\bar{\kappa }\left(s\right)KdA$ is dependent on the geometry of the boundary line and no longer a topological invariant. In the context of multi-component lipid bilayers, this term has been shown to contribute to the formation of vesicles [14–18]. A Gaussian curvature modulus can in principle have either sign. For lipid bilayers ${\bar{\kappa }}_{L}$ is known to be negative and of the order of the bending modulus −*κ*_*L*_ [25]. In order for the minimum of the Helfrich bending energy to correspond to a mechanically stable spherical cap, the Gaussian curvature modulus ${\bar{\kappa }}_{C}$ of the capsid also should be negative and in the range $-2\kappa_{C}<{\bar{\kappa }}_{C}<0$ (in the limit that ${\bar{\kappa }}_{C}=0$, the spherical cap shape is marginally stable).

**Fig 3:**
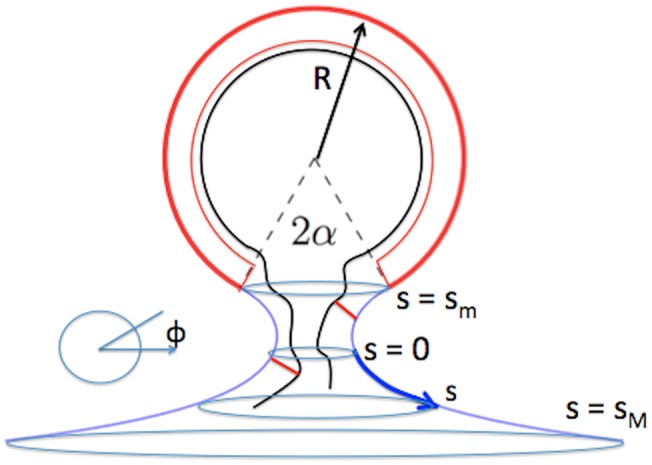
Shape of a bud that minimizes the Helfrich bending energy. The blue line, which represents the bare lipid bilayer membrane, has the shape of catenoid of revolution. The heavy red line, which represents the lipid bilayer attached to a curved layer of capsid proteins, has the shape of spherical cap. The interface is a circle. The boundary between the two bilayers and the center of the sphere spans a cone with aperture angle 2*α*. For purposes of illustration, RNA genome molecules associated with the bud are indicated by a black line. Two capsid proteins diffusing along the lipid bilayer are indicated as two red bars associated both with the membrane and an RNA genome molecule. A curvilinear coordinate system (*s*, *φ*) is indicated where *s* measures the shortest arc distance between a point and the cross-section with minimum diameter (s = 0). The value of *s* ranges from *s*_*M*_ > 0 to *s*_*m*_ < 0. Finally, *φ* is the azimuthal angle of the circle on the surface perpendicular to the central axis on which the point is located.

**Fig 4:**
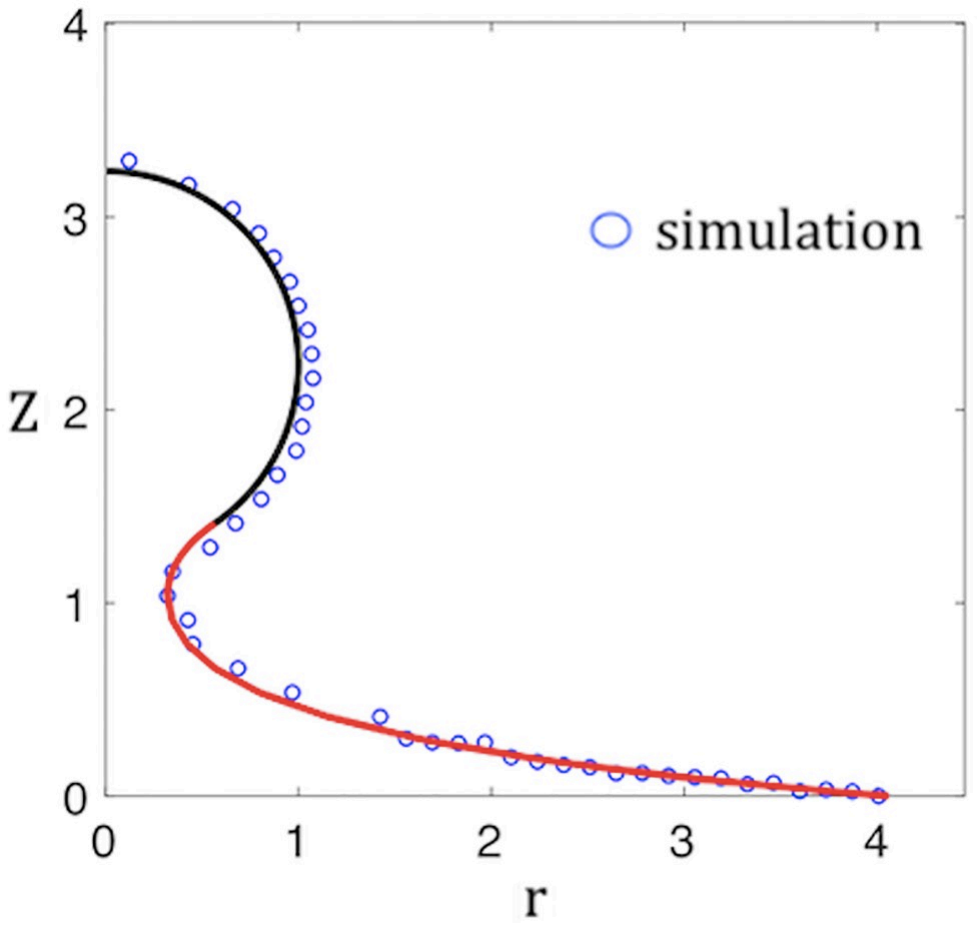
Comparison between the shape of the bud obtained from the simulations (open circles) and the one that minimizes the Helfrich bending energy. The latter is composed of a spherical cap (black line) joined to a catenoid of revolution (red line). Vertical axis: height Z in arbitrary units. Horizontal axis: radial distance r in arbitrary units. The aperture angle *α* is treated as a fitting parameter.

Under conditions of mechanical equilibrium, the Helfrich energy must be minimized. A budding geometry that minimizes the Helfrich bending energy is shown in Fig 3. A spherical cap shape, which represents the partially assembled capsid, is attached to a *minimal surface* shape (i.e., a surface with H = 0) in the form of a catenoid of revolution. This represents the protein-free lipid bilayer. The interfacial boundary joining the two parts is a circle. The aperture angle of the cone subtended by the center of the sphere and the boundary line will be denoted by *α*. If the area *A* of the spherical cap equals *A* = *πρ*^2^ then there is a geometrical relation between *ρ* and the aperture angle given by *ρ* = 2*R* cos *α*/2. Fig 4 compares the shape of the profile obtained from the simulations with this shape, with *α* treated as a fitting parameter. The agreement is reasonable but the theory does not reproduce a certain amount of large-scale warping of the membrane that was observed during the early stages of budding.

The total continuum energy is the sum of the Helfrich bending energy of the capsid and the membrane, an interfacial free energy *τP*, with *P* the length of the interfacial boundary with *τ* the interfacial energy per unit length. The last term is the cohesion energy −*σA*, with *σ* the free energy gain per unit area for capsid assembly. One can show that, for given area *A*, the total continuum energy can be expressed as a dimensionless function of the aperture angle *α*:

$$\begin{array}{c} F\left(\alpha \right)/\kappa_{C}=2\pi {\left(2\;\text{cos}\frac{\alpha }{2}-\rho \right)}^{2}-4\pi \bar{g}{\;\text{cos}}^{2}\frac{\alpha }{2}+2\pi \tau \rho \text{sin}\;\alpha /2-\pi \sigma \rho^{2} \end{array}$$ (3)

We redefined *ρ* as *ρ*/*R*_0_, a dimensionless growth parameter; *τ* as *τR*_0_/*κ*_*C*_, a dimensionless line energy per unit length; and *σ* as $\sigma R_{0}^{2}/\kappa_{C}$, a dimensionless cohesion energy per unit area. The first term corresponds to the mean or “extrinsic” curvature term of the Helfrich free energy while the second second term, with $\bar{g}=\frac{\left(|{\bar{\kappa }}_{C}|-|{\bar{\kappa }}_{L}|\right)}{\kappa_{C}}$, corresponds to the Gaussian or “intrinsic” curvature energy. Note that $\bar{g}$ vanishes when the Gaussian curvature moduli of the capsid and the bare lipid bilayer are the same. It is expected that $\bar{g}$ is positive because the bending moduli of the capsid are expected to be larger in magnitude than those of the lipid bilayer. The form for the Gaussian curvature energy follows from an application of the Gauss-Bonnet Theorem. The last two terms are the cohesion and interfacial line energy terms respectively.

**Fig 5:**
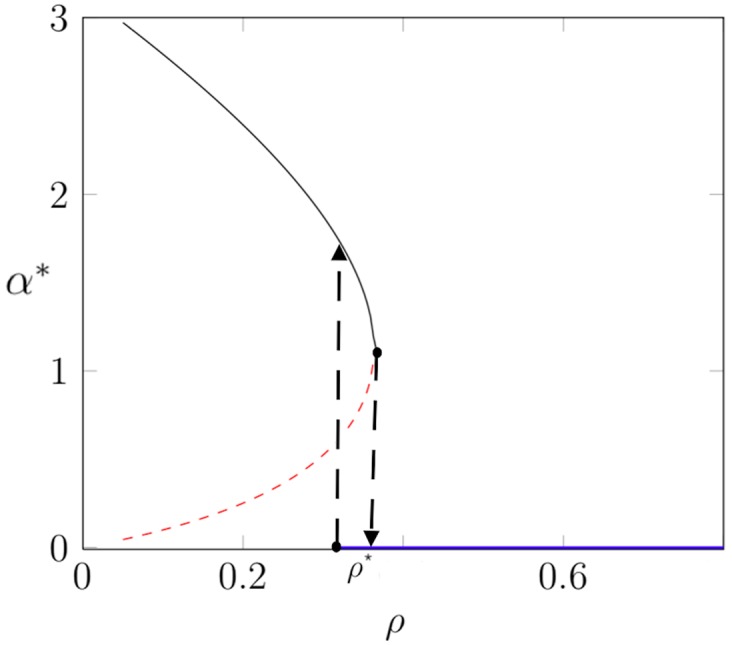
Example of the dependence of the aperture angle α* that minimizes the free energy on the growth parameter *ρ* for the case of a fluyid shell. The partial shell in the shape of a spherical cap with α* > 0 is locally stable along the black line and unstable along the red dashed line. The completed capsid with α* = 0 is stable along the blue line. The black dashed lines mark limits of local stability. Parameter values: *κ*_*C*_ = 0.5, *σ* = 0, $\bar{g}=1.4$, and *τ* = 0.5.

The next step depends on whether the capsid shell is to be treated as an ordered particle array or as a fluid (or visco-elastic) system. First consider the fluid case. If the capsid shell is fluid, then *α* can be treated as a variational parameter to be determined by free energy minimization. The function *F*(*α*) has two minima: one at a non-zero *α* = α*(*ρ*) that corresponds to an open spherical cap and one at *α* = 0 that corresponds to a closed shell. A typical example of the dependence α*(*ρ*) is shown in Fig 5. As *ρ* increases from zero, α*(*ρ*) decreases monotonically from α*(0) = *π*. Initially, the spherical cap state is the minimum free energy state, but at a point *ρ* = ρ*, the energy of the *α* = 0 closed shell state drops below that of the *α* = α*(*ρ*) spherical cap state. At that point, the spherical cap state is connected via a neck to the membrane. At a slightly larger value of *ρ*, the spherical cap state becomes *locally unstable* and abruptly transforms at fixed *ρ* to a closed shell state. Within continuum theory, no energy barrier prevents this transformation.

**Fig 6:**
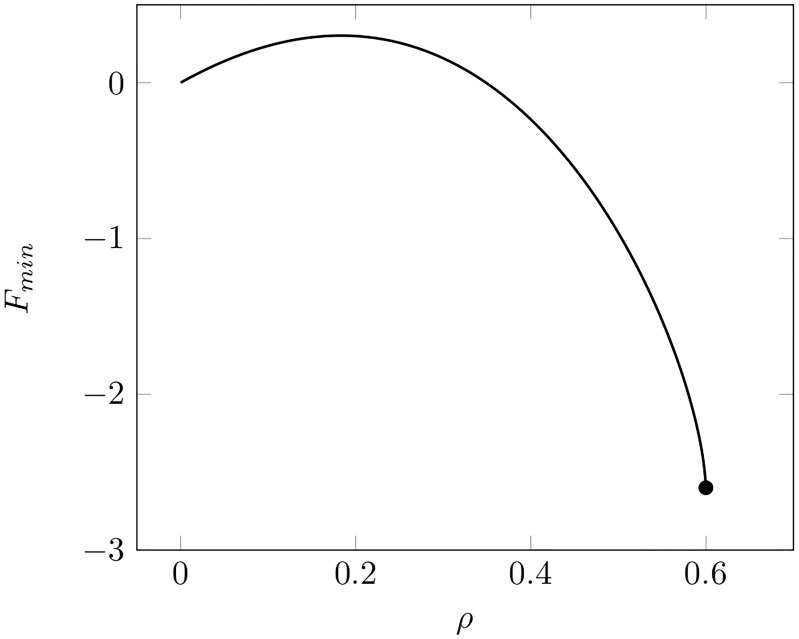
Example of the dependence of the free energy minimum on the growth parameter *ρ* for the case of a solid shell. The parameters were the same as for Fig 5 while the cohesion energy parameter *σ* was set to zero. The dot indicates the point where the neck diameter has shrunk to zero. Note that there is an activation barrier for low values of the growth parameter but not at the point of pinch-off.

If, on the other hand, a capsid shell is a positionally ordered solid then an *elastic strain energy* term must be included in the free energy. A local change of the Gaussian curvature of an ordered layer generates large elastic stresses [26–28]. In consequence, *if* a growing bud always has the shape of a spherical cap then the radius of curvature must stay the same during the growth process (or nearly so) since changing the curvature radius means changing the Gauss curvature. In particular, a discontinuous transformation from an open to a closed shell at fixed total area, such as encountered for the fluid shell, is not possible for a positionally ordered shell. Assuming thus the curvature radius *R* to be a fixed quantity, the aperture *α* and the growth parameter *ρ* are related by the condition *ρ* = 2*R* cos *α*/2 so *α* is no longer a variational parameter. A plot of *F* for fixed *R* as a function of *ρ* is shown in Fig 6. In the first part of the curve an activation barrier can be seen but this barrier is a standard feature of the nucleation and growth process. In the second part of the plot, where the neck is forming, the free energy decreases monotonically for increasing growth parameter. Increasing the cohesion energy from zero only facilitates the budding.

This way of including the elastic energy is only heuristic. In order to verify it, we again used our Brownian dynamics simulations of alphavirus budding. The proteins of the shell were, in this case, positionally ordered and had a curvature radius that did not change significantly during budding. The elastic shell description is then the appropriate one. In order to check for the effects of strain, the simulations were repeated *in the absence of a lipid bilayer*, with monomers in solution freely diffusing to the edge of the growing shell. The capsid shape and the degree of positional ordering remained similar, so the elastic strains presumably also were not much affected by the removal of the bilayer. Yet the pausing and stalling effects completely disappeared. We conclude that pausing/stalling is a feature of the membrane geometry that it is not related to curvature-induced elastic stresses.

One important caveat is that the the Helfrich description is not a good description of the membrane during the scission process itself, when the connection between the virus and the membrane has reduced to an elongated stalk. At the very least, a continuum description of scission needs to include the bilayer nature of the membrane along the lines of the continuum theory for stalk formation during membrane fusion [29]. It is certainly possible that an energy barrier could appear in such a theory. However, the stalling observed in the simulations happened when the opening in the membrane still was quite large compared to the thickness of a lipid bilayer so the growth of an energy barrier associated with stalk formation is not the explanation for the stalling observed in the simulations.

### Continuum theory: Surface diffusion

Since we could find no energy barrier preventing scission within equilibrium continuum theory, could the stalling and pausing be a purely kinetic effect? In other words, could it be an effect involving the transport of capsid proteins to the growing shell? For the case of the alphavirus simulations, the transmembrane capsid glycoproteins were localized to the PM. In order to construct a continuum theory for the dynamics, we will focus on the case that the transport of capsid proteins to the growing bud proceeds by surface diffusion along the PM to the growing bud. The proteins are incorporated along the growth interface, which acts as a protein absorber.

The theory of diffusive transport of membrane inclusions along nearly flat membranes has been extensively studied (see ref. [30] and references therein), which has been extended to diffusive transport on curved surfaces [31–33]. What is relevant for the present case is the fact that the diffusion coefficient on surfaces with negative Gauss curvature is *larger* than the diffusion coefficient of the same inclusion on a corresponding flat surface [31], which hardly seems encouraging. However, these references focused on membrane inclusions that did not couple to the curvature of the membrane. As discussed earlier, stability of the spherical caps shape requires that the capsid proteins must be coupled to Gauss curvature. In fact, their chemical potential should be minimized for positive Gauss curvature. In the following, we will explore how curvature coupling alters the physics of membrane diffusive transport of proteins.

To develop a continuum description for diffusive transport, introduce a low concentration *ϕ* of membrane-associated capsid protein monomers or oligomers that are diffusing in to the partial shell. We retain the geometry of Fig 3 with *R* = *R*_0_. The first step in computing the steady-state diffusion current from infinity to the circular interface between the capsid and the lipid bilayer is to introduce a curvilinear coordinate system (*s*, *φ*) along the catenoid of revolution shown in Fig 3. Here, ***s*** measures the arc distance of a point on the surface to the minimum cross-section of the neck (at s = 0) while *φ* measures the azimuthal angle. Assuming that the steady-state current has rotational symmetry, the surface protein concentration *ϕ*(*s*) in the neck region should only depend on *s*. Far from the neck area, the capsid protein concentration is set equal to a constant value *ϕ*_0_. When a diffusing capsid protein arrives at the circular interface, it is assumed to be immediately absorbed into the shell, which means that *ϕ* = 0 at the growth interface (“absorber” boundary condition).

According to the principles of non-equilibrium thermodynamics, if interaction between the capsid proteins is neglected then the current density *J*^*i*^ of the capsid proteins, with *i* a component of the curvilinear coordinate system of Fig 3, is proportional to the gradient of the chemical potential along the curved surface. In the limit of low concentrations, this chemical potential gradient can be expressed as

$$\begin{array}{c} J^{i}=-D\nabla^{i}\phi -\mu \phi \nabla^{i}\epsilon \end{array}$$ (4)

The first term is the gradient of the contribution to the chemical potential coming from translational entropy with *D* the surface diffusion coefficient. In the second term, the drift term, the mobility *μ* is related to the diffusion coefficient through Einstein’s relation *μ* = *D*/*k*_*B*_*T*. The second term is the gradient of the enthalpic contribution *ϵ*(*s*) to the chemical potential. This quantity can be obtained from the relation $\epsilon =\frac{\delta E([\phi )]}{\delta \phi }$ where *E*([*ϕ*)] is the enthalpic part of the free energy of the lipid bilayer. By repeating the symmetry arguments that are used to obtain the Helfrich bending energy for a curved membrane [19], it follows that *E*([*ϕ*)] must have the general form

$$\begin{array}{c} E\left(\left[\phi \right]\right)=E_{0}\left(\left[\phi \right]\right)+\;\int_{A}\left[2\kappa \left(\phi \right){\left(H-C\left(\phi \right)\right)}^{2}+\bar{\kappa }\left(\phi \right)K\right]dA, \end{array}$$ (5)

where *E*_0_([*ϕ*]) is the protein-membrane interaction energy of a flat membrane while *κ*(*ϕ*) and $\bar{\kappa }\left(\phi \right)$ are the concentration-dependent curvature moduli. Finally, *C*(*ϕ*) is the concentration-dependent spontaneous curvature. In the Materials and Methods section we show that in the small *ϕ* limit, $\epsilon \left(s\right)={\bar{\kappa }}^{\prime }K\left(s\right)$ where ${\bar{\kappa }}^{\prime }$, the derivative of $\bar{\kappa }\left(\phi \right)$ with respect to *ϕ* at *ϕ* = 0, is a negative quantity.

The steady-state current is the solution of the continuity equation

$$\begin{array}{c} \nabla_{i}J^{i}=\frac{1}{\sqrt{g}}\partial_{i}\left(\sqrt{g}J^{i}\right)=0 \end{array}$$ (6)

where *g* is the determinant of the *metric tensor* of the surface. The solution must obey the condition that the capsid protein concentration is zero just outside the circular interface (absorber boundary condition) while far from the interface it should approach the prescribed surface area concentration. The mathematical method that was used in solving the continuity equations is straightforward and discussed in Materials and Methods. The current will be expressed in terms of the maximum current and while the growth parameter *ρ* will be expressed in terms of the maximum value *ρ*_*M*_. This maximum value is defined by the condition that the area *πρM*^2^ of a flat disk of protein material equals the surface area $4\pi R_{0}^{2}$ of the capsid. The relation between current and growth parameter is controlled by the single dimensionless quantity $\gamma =\beta |{\bar{\kappa }}^{\prime }|/R_{0}^{2}$.

**Fig 7:**
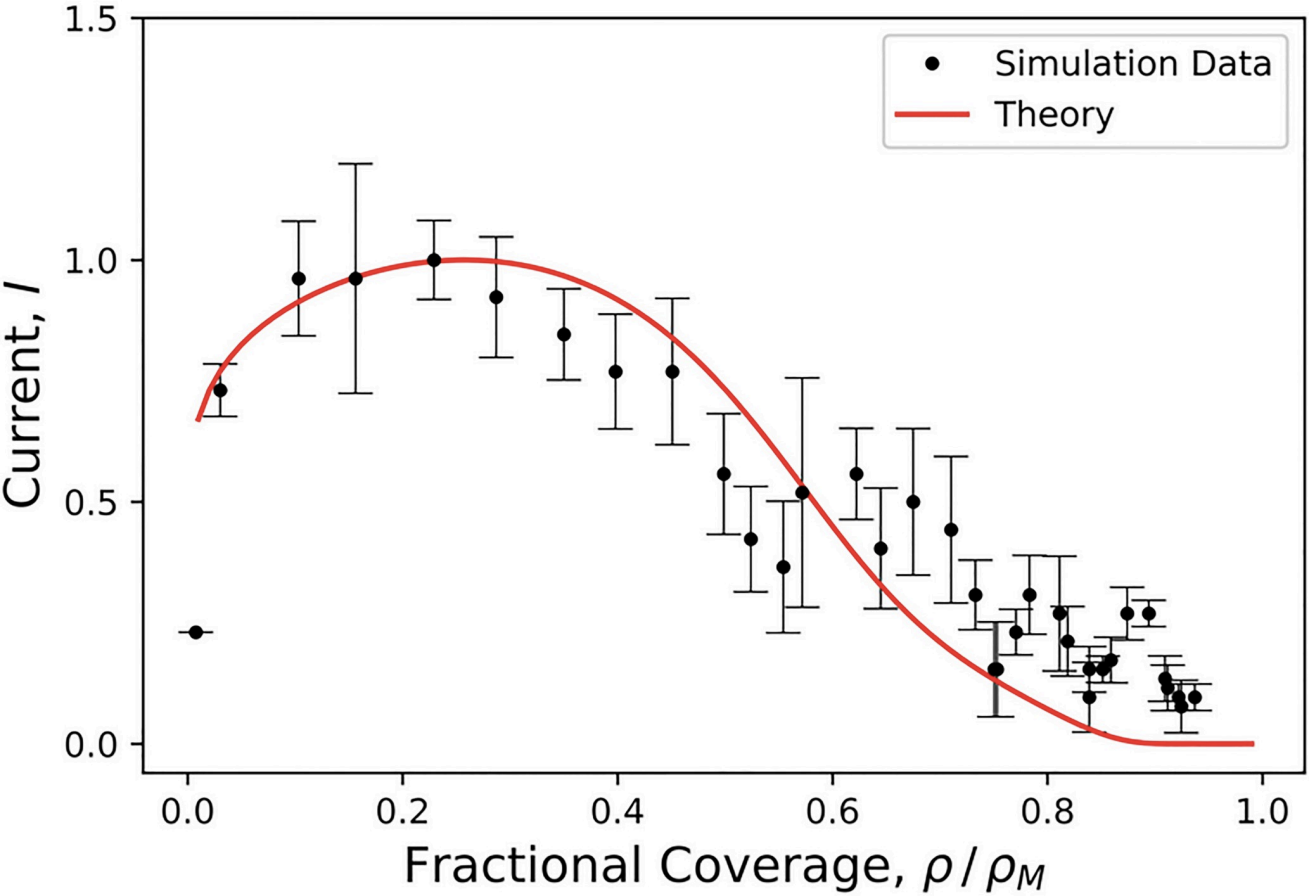
Solid red line: Diffusion current *I* from the exterior to the growing bud versus relative size *ρ*/*ρ*_*M*_ of the bud computed from Eq 17. *γ* = 0.9 was the sole fitting parameter. The current and the growth parameter were normalized with respect to their maximum values. Black dots: assembly current obtained from the Brownian Dynamics simulation of Fig 2. Error bars were obtained by averaging over three runs. The strength *ϵ*_gg_ of the interaction between the capsid proteins was 6*k*_B_*T*. The black arrow indicates the location of the maximum of the current profile predicted by conventional diffusive transport theory (i.e. *γ* = 0 in Eq 11).

Fig 7 shows a comparison between a plot of the relation between current and growth parameter with the outcome of the simulations of Fig 2. The *γ* parameter was used as a fitting parameter. Eq 17 (red line) is reasonably consistent with the data, but it underestimates the current across the neck just prior to pinch-off. A key feature is the maximum of both the computed data and Eq 17. The presence of a maximum in the *I* − *ρ* profile is a feature of conventional diffusive transport with *γ* = 0 on a flat surface, as was noted by a number of authors [34–37]). However, for *γ* = 0 the maximum is at $\rho /\rho_{M}=1/\sqrt{2}$ when the partial shell has the shape of a hemisphere. This point is denoted by an arrow in the figure. The position of that maximum disagrees strongly with the maximum in data of Fig 7, so we definitely can rule out conventional diffusive transport theory. An important difference between the measured data and Eq 17 is that the measured assembly current actually does not go to zero: the theory appears to overestimate the geometrical barrier for small neck widths.

While the theory is consistent with the data, the comparison is not conclusive. We compared the concentration profiles of the theory and the simulations in the neck region, to obtain additional verification, but the statistical error due to the small number of proteins in the neck region and the out-of-equilibrium nature of the simulations was too large for a meaningful comparison.

### Conclusions

In summary, the physics of diffusion of Gaussian curvature-sensing proteins provides us with a mechanism that could explain the pausing and stalling that is observed during the late-stage budding of many enveloped viruses. This mechanism is based on the fact that capsid proteins diffusing in from infinity towards the growth interface of a viral bud necessarily must pass through a neck region with negative Gauss curvature. The chemical potential of the capsid proteins is increased in the neck region since capsid proteins intrinsically impose positive Gauss curvature on the PM of the host cell. Within a simple continuum theory, the importance of this geometrical barrier effect is determined by a dimensionless parameter, $\gamma =\beta |{\bar{\kappa }}^{\prime }|/R_{0}^{2}$. If *γ* is of the order of one or larger, then the suppression of the current by the geometrical barrier shows up already at relatively large aperture angles while for smaller *γ*, the effect appears only for increasingly smaller apertures. One can estimate *γ* if one assumes that ${\bar{\kappa }}^{\prime }\phi_{M}$ is of the order of the Gauss curvature modulus ${\bar{\kappa }}_{C}$ of the capsid. Here, *ϕ*_*M*_ ≃ 1/*a*^2^ is the protein concentration of the capsid with *a* of the order of a nanometer. Next, numerical estimates of Gauss moduli typically produce $\bar{\kappa }≃-\kappa$ [38, 39]. Assuming a curvature modulus of viral capsids in the range of 100 *k*_*B*_*T*, one finds *γ* values in the range of 0.1. The dependence of the growth rate on the amount of capsid material measured for numerical simulations of the assembly of the alphavirus indeed can be fitted by values of *γ* in this range.

There are however also disagreements between theory and simulations. Within the continuum theory, the budding process should actually come to a complete halt but the numerical simulations demonstrate that in general this is not the case. There appears to be a critical point in terms of the protein-protein interaction strength *ϵ*_gg_ below which assembly indeed stalls but above which budding completes in a spontaneous scission event. A continuum theory that takes into account the bilayer nature of the membrane and the nature of the interaction between the lipid bilayer and capsid proteins may be required to explain the fact that scission eventually does take place.

If scission is spontaneous for sufficiently large values of *ϵ*_gg_, then why do enveloped viruses not adopt this route? It would avoid the necessity of having to recruit the ESCRT machinery. One reason could be that, according to the numerical simulations, the capsid becomes increasingly *defected* for large *ϵ*_gg_, which could interfere with other functions of the virus. Another manner in which the geometrical barrier could be suppressed would be if capsid proteins coupled only to the mean curvature and not to the Gaussian curvature. Because the spherical cap shape becomes mechanically unstable when $\bar{\kappa }$ goes to zero, this also may not be an option. The opportunistic recruitment of the ESCRT cell machinery—which has been referred to as a “Trojan Horse” strategy [40]– avoids these problems.

Next, we have assumed in this paper that the pausing is not *caused* by the ESCRT machinery. A reconstruction of the formation of the ESCRT machinery during HIV budding [8] shows that the ESCRT machinery occupies a significant amount of space in the neck region, which suggests the possibility that the ESCRT machinery prevents diffusing capsid proteins from reaching the growth surface. To us this explanation appears to be less likely. First, the immature capsid of HIV has holes in multiple locations [3] while templated self-assembly of Gag proteins in the absence of ESCRT [41] produces capsids with holes. The tendency to form holes is thus an intrinsic feature of the HIV capsid instead of a by-product of the ESCRT machinery. Next, the ESCRT machinery is recruited also during the budding of enveloped viruses that have completely closed proteins shells, such as Herpes Simplex. In those cases apparently, ESCRT is not blocking protein transport completion of the protein shell. Finally, if the ESCRT machinery causes stalling then why would it be recruited in the first place? It is more parsimonious to assume that the ESCRT machinery does not block assembly and that pausing/stalling has a separate cause. The striking difference between the HIV and Herpes capsids is interesting in its own right. It will have to be understood in terms of differences between the interactions between the respective capsid proteins. The capsid proteins of Herpes—and those of the related bacteriophage viruses such HK97—are highly evolved “molecular machines” that weave together ordered and robust shells [42]. On the other hand, the defected nature of the immature HIV capsid [3] suggest that the interactions are relatively weak in that case. Within our model simulations, the strength of the attractive interactions between the model proteins would be the only handle to account for such differences. Interestingly, though, increasing the strength of the attractive interactions does cause the holes to disappear.

An important limitation of the transport theory that we presented is that it assumes that the diffusing capsid proteins are non-interacting. It is in fact known that there are long-range, membrane-mediated interactions between membrane-associated proteins that apply a bending moment to the membrane [43]. These interactions can stimulate aggregation and also produce multiple budding events in a concerted fashion as discussed by Auth and Gompper [44] and by Reynwar et al. [45]. The model proteins of our simulations do apply such a bending moment but only when they form oligomers. It would be interesting to investigate how such interactions affect the geometrical kinetic barrier that is causing the stalling (see ref. [30]).

Finally, it is interesting to compare the budding of enveloped viruses with cellular endocytotic processes that involve a member of the BAR-domain familiy of membrane-associating proteins, which are banana-shaped curvature-sensing proteins. At high densities, BAR-domain proteins are able to control membrane shape by inducing membrane curvature. This can lead to the formation of membrane tubules [46]. Tubular membranes surrounded by BAR-domain proteins associate with dynamin [47], which produces scission. Like the BAR-domain proteins, the capsid proteins of enveloped viruses have an affinity for membranes, but exclusively for membranes with positive Gauss curvature and, unlike BAR-domain proteins, capsid proteins avoid membrane section with negative Gauss curvature, which is what led to the geometrical barrier. Replacing capsid proteins by BAR-proteins, presumably tranforms the neck to a *potential well*. It would be interesting to explore the effects of BAR-proteins on the budding of enveloped viruses.

## Materials and methods

**Fig 8:**
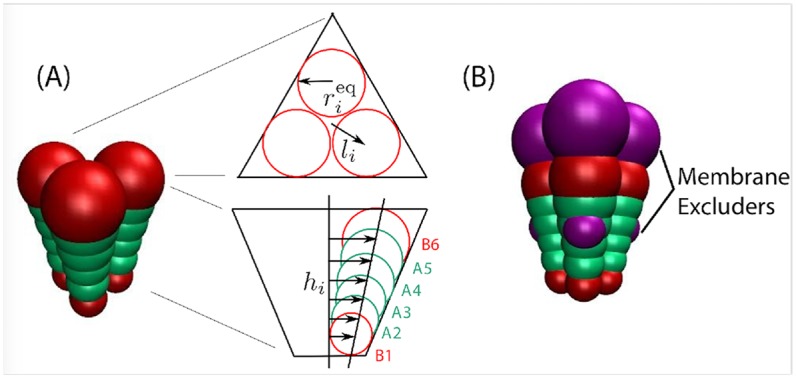
**(A)** (left) Image of a trimer subunit, with attractors (‘A_2_’-‘A_5_’) in green and excluders (‘B_1_’ and ‘B_6_’) in red. (right) Schematic of the subunit geometry, with views from directly above the plane of the membrane and within the plane of the membrane. Membrane excluders are not shown in these schematics to aid visual clarity. **(B)** Image of a subunit trimer, showing attractors (green, type ‘A’), excluders (red, type ‘B’), and membrane excluders (magenta, type ‘VX’).

Our simulations employ a coarse-grained model that was designed to capture the essential physical features of the membrane and alphavirus transmembrane glycoproteins (GPs) (see Fig 8). Although the GPs are transmembrane proteins, their assembly is described by the same continuum model as HIV capsid proteins adsorbed to the membrane. Moreover, in our model the conical regions which drive curvature of the model subunit oligomers are located within and below the plane of the membrane, as we found that this arrangement facilitated completion of assembly [1]. We note that the stalling described in the main text was observed for all subunit interaction geometries that we have considered for the alphavirus model [1], as well as in another model for proteins that adsorb onto the membrane [48], suggesting that the barrier is a generic feature of assembly and budding on a membrane. We note that the stalling described in the main text was not observed in some previous budding simulations because they only considered early stages of budding [49–51]. While Refs. [52, 53] did consider the entire budding process, their model represented the capsid proteins as patchy spheres and the membrane as a triangulated monolayer, which likely eliminated or minimized coupling of the proteins to membrane Gaussian curvature.

We begin with an overview of each component of the model, and then give the full set of interaction potentials in section.

### Glycoproteins and capsid

Our model GPs are designed to roughly match the triangular shape, dimensions and aspect ratio of trimers-of-heterodimers of E1 and E2 GPs in the Sindbis virion [1, 54, 55]. There are 80 of these trimers arranged with T = 4 icosahedral symmetry in the virion structure. On the capsid surface each trimer forms a roughly equilateral triangle with edge-length ∼ 8nm. In the radial direction, each E1-E2 heterodimer spans the entire lipid membrane and the ectodomain spike, totaling ∼ 12nm in length.

Our GP trimer subunit comprises three cones, which are fused together and simulated as a rigid body. Each cone is represented by an array of 6 beads of increasing diameter, following the model described by Chen et al. [56]. However, our cones are truncated, so that they form a shell with an empty interior, as shown in Fig 8. The cones experience lateral interactions, with a preferred angle that, in the absence of a membrane, drives assembly into shells with a typical size of 80 trimers, with fluctuations in the range 79 − 82 trimers. The attractions are mediated by the four interior pseudoatoms within each cone (A2-A5 in Fig 8), while the innermost and outermost pseudoatoms (B1 and B6) experience only excluded volume.

### Lipid membrane

The lipid membrane is represented by the implicit solvent model from Cooke and Deserno [13]. This model enables, on computationally accessible timescales, the formation and reshaping of bilayers with physical properties such as rigidity, fluidity, and diffusivity that can be tuned across the range of biologically relevant values. Each lipid is modeled by a linear polymer of three beads connected by FENE bonds; one bead accounts for the lipid head and two beads for the lipid tail. An attractive potential between the tail beads represents the hydrophobic forces that drive lipid self-assembly. For the simulations described here, the membrane bending modulus was set to *κ*_mem_ ≈ 12.5*k*_B_*T*.

### Glycoprotein-membrane interactions

We use a minimal model for the GP-membrane interaction. We add six membrane excluder beads (type ‘VX’) to our subunit, three at the top and three at the bottom of the subunit, with top and bottom beads separated by 7nm (magenta beads in Fig 8B). These excluder beads interact through a repulsive Lennard-Jones potential with all membrane beads, whereas all the other cone beads do not interact with the membrane pseudoatoms. In a simulation, the subunits are initialized with membrane located between the top and bottom layer of excluders. The excluded volume interactions thus trap the subunits in the membrane throughout the length of the simulation, but allow them to tilt and diffuse laterally.

### GP conformational changes and implementation of constant GP concentration

Experiments suggest that viral proteins from many families interconvert between ‘assembly-active’ and ‘assembly-inactive’ conformations, which are respectively compatible or incompatible with assembly into the virion [57–59]. Experiments suggest similar conformational changes for the alphavirus GPs E1 and E2 [59, 60]. Computational modeling suggests that such conformational dynamics can suppress kinetic traps [61, 62]. Based on these considerations, our GP model includes interconversion between assembly-active and assembly-inactive conformations. The two conformations have identical geometries, but only assembly-active conformations experience attractive interactions to neighboring subunits. I.e., there are no attractive interactions (Eq 12 below) for subunit pairs in which one or both of the subunits is in the inactive conformation.

We adopt the ‘Induced-Fit’ model of Ref. [61], meaning that interaction with an assembling GP shell or the NC favors the assembly-active conformation. For simplicity, we consider the limit of infinite activation energy. In particular, with a periodicity of *τ*_c_ all the inactive subunits found within a distance 1.0*σ* of the capsid are switched to the active conformation, while any active subunits further than this distance from an assembling shell convert to the inactive conformation. Results were unchanged when we performed simulations at finite activation energies larger than 4*k*_B_*T*.

To maintain a constant subunit concentration within the membrane (outside of the region where an assembling shell is located) we include a third subunit type called ‘reservoir subunits’, which effectively acts as a reservoir of inactive subunits. These subunits interact with membrane beads but experience no interactions with the other two types of GP subunits. With a periodicity of *τ*_c_, reservoir subunits located in a local region free of active or inactive subunits (corresponding to a circumference of 1.5 times the radius of the largest subunit bead) are switched to the assembly-inactive state.

### Subunit geometry

The geometry of the model GP trimer subunit is schematically shown in Fig 8. As explained above, the subunit consists of three cones symmetrically placed around the subunit axis. Each cone contains six pseudoatoms. Only the inner four pseudoatoms (denoted as A) experience attrative interactions. The outer two pseudoatoms, B, interact with the rest through excluded volume. The pseudoatoms are placed at heights *h*_*i*_ = [16.0, 17.5, 19.0, 20.5, 22.0, 23.5]*σ*. At each plane *z* = *h*_*i*_ there are three identical pseudoatoms forming an equilateral triangle of radius *l*_*i*_ = *h*_*i*_ tan *α*_*l*_, where *α*_*l*_ can be tuned. Since assembly in bulk is slightly more robust for smaller *α*_*l*_, we choose an optimal value *α*_*l*_ = 7°. The radius of each pseudoatom is then given by $r_{i}^{\text{eq}}=l_{i}\cos\psi$, with *ψψ* = 94.9° the parameter that controls the preferred curvature of the subunits. Finally, to embed the subunits in the membrane we add two layers of three membrane excluders ‘VX’, consistent with the cone geometry, at height *h*_in_ = 19.0*σ* (inner domain) and *h*_out_ = 26.0*σ* (outer domain). The sequence of pseudoatoms across the shell reads [B_1_,A_2_,A_3_,VX_in_,A_4_,A_5_,B_6_,VX_out_].

### Simulations

We perform simulations in HOOMD-blue [63], version 1.3.1. Both the subunits and the NC are simulated using the Brownian dynamics algorithm for rigid bodies. The membrane dynamics is integrated using the NPT algorithm, a modified implementation of the Martina-Tobias-Klein thermostat-barostat. The box size changes in the membrane plane, to allow membrane relaxation and maintain a constant lateral pressure. The out-of-plane dimension is fixed at 200*σ*.

Our simulations use a membrane patch with size 170 × 170nm^2^ (*A* ∼ 28,900nm^2^), which contains 51,842 lipids. We compare membrane deformations, capsid size and organization from these simulations against a set of simulations on a larger membrane (210 × 210nm^2^, *A* ∼ 44,100nm^2^) and observed no significant differences, suggesting that finite size effects were minimal. Simulations are initialized with 160 subunits uniformly distributed on the membrane, including 4 active-binding subunits (located at the center of the membrane) with the remainder in the assembly-inactive conformation. In addition, there are 156 subunits in the reservoir conformation uniformly distributed.

The membrane is then equilibrated to relax any unphysical effects from subunit placement by integrating the dynamics for 1,500 *τ*_0_ without attractive interactions between GPs. Simulations are then performed for 4,200 *τ*_0_ with all interactions turned on. The timestep is set to Δ*t* = 0.0015, and the thermostat and barostat coupling constants were *τ*_*T*_ = 0.4 and *τ*_*P*_ = 0.5, respectively. Since the tension within the cell membrane during alphavirus budding is unknown, we set the reference pressure to *P*_0_ = 0 to simulate a tensionless membrane. The conformational switching timescale is set to *τ*_c_ = 3*τ*_0_, sufficiently frequent that the dynamics are insensitive to changes in this parameter.

### Interaction potentials

The total interaction energy *U*_tot_ is separated into two contributions,

$$\begin{array}{c} U_{\text{tot}}=U_{\text{mem}}+U_{\text{gg}} \end{array}$$ (7)

where *U*_mem_ represents the interaction energy between the membrane beads and *U*_gg_ accounts for the interaction of between subunits as well as with the membrane.

#### Membrane interactions

The membrane lipids consist of three beads, the first representing the lipid head and the other two connected through two finite extensible nonlinear elastic (FENE) bonds with maximum length *r*_cut_ = 1.5*σ*,

$$\begin{array}{c} U_{\text{bond}}\left(r\right)=-\frac{1}{2}k_{\text{bond}}r_{\text{cut}}^{2}\log\left[1-{\left(r/r_{\text{cut}}\right)}^{2}\right]. \end{array}$$ (8)

with *k*_bond_ = 30*ϵ*_0_/*σ*^2^. A harmonic spring links the two outer beads, to ensure that the lipids maintain a cylindrical shape,

$$\begin{array}{c} U_{\text{bend}}\left(r\right)=\frac{1}{2}k_{\text{bend}}{\left(r-4\sigma \right)}^{2}. \end{array}$$ (9)

All membrane beads interact via a Weeks-Chandler-Andersen potential,

$$\begin{array}{c} U_{\text{rep}}\left(r\right)=\sum^{\;}4\epsilon_{\text{rep}}\left[{\left(\frac{b_{i,j}}{r}\right)}^{12}-{\left(\frac{b_{i,j}}{r}\right)}^{6}+\frac{1}{4}\right], \end{array}$$ (10)

with *ϵ*_rep_ = 1 and cutoff *r*_cut_ = 2^1/6^ *b*_*i*,*j*_. The parameter *b*_*i*,*j*_ depends on the identities of the interacting beads: *b*_h,h_ = *b*_h,t_ = 0.95*σ* and *b*_t,t_ = 1.0*σ*, with the subscripts ‘h’ and ‘t’ denoting head and tail beads, respectively. The hydrophobic nature of the lipid tails is accounted for by an attractive interaction between all pairs of tail beads:

$$\begin{array}{c} U_{\text{hydro}}\left(r\right)=\{\begin{array}{cc} -\epsilon_{0}, & r<r_{\text{c}}\\ -\epsilon_{0}\cos\left[\pi \left(r-r_{\text{c}}\right)/2\omega_{\text{c}}\right], & r_{\text{c}}\leq r\leq r_{\text{c}}+\omega_{\text{c}}\\ 0, & r>r_{\text{c}}+\omega_{\text{c}} \end{array} \end{array}$$ (11)

with *ϵ*_0_ = 1.0, *r*_c_ = 2^1/6^*σ*. The potential width *ω*_c_ is a control parameter that determines, among other properties, the membrane rigidity. Unless otherwise specified, *ω*_c_ = 1.6.

#### GP-GP interactions

The interaction potential between pairs of GP subunits, *U*_gg_, consists of two terms. If both subunits are in the active conformation, there is an attractive interaction between pairs of attractor pseudoatoms ‘A’, modeled by a Morse potential. Beads interact only with those of the same kind on a neighboring cone, A_*i*_-A_*i*_, *i* = 2,.., 5, and the equilibrium distance of the potential depends on the pseudoatom radius, $r_{i}^{\text{eq}}$:

$$\begin{array}{c} U_{\text{gg}}^{M}=\sum_{i=2}^{5}U_{i}^{M}=\sum_{i=2}^{5}\epsilon_{\text{gg}}\left(e^{-2\alpha_{i}\left(r-2r_{i}^{\text{eq}}\right)}\right)-2e^{-\alpha_{i}\left(r-2r_{i}^{\text{eq}}\right)}) \end{array}$$ (12)

with $\alpha_{i}=\left(3.0/r_{i}^{\text{eq}}\right)$. The cutoff of this interaction was set at $r_{\text{cut}}=2r_{i}^{eq}+3.5$. All subunit beads of type ‘A’ and ‘B’ experience excluded volume interactions regardless of whether subunits are in the active or inactive conformations, according to:

$$\begin{array}{c} U_{\text{g-g}}^{\text{ex}}\left(r\right)=\sum_{i}\sum_{j}4\epsilon_{\text{ex}}\left[{\left(\frac{b_{i,j}}{r}\right)}^{12}-{\left(\frac{b_{i,j}}{r}\right)}^{6}\right] \end{array}$$ (13)

with *ϵ*_ex_ = 1.0 and cutoff radius $r_{\text{cut}}=b_{ij}=r_{i}^{\text{eq}}+r_{j}^{\text{eq}}$. The sum extends to all the subunit beads of type ‘A’ and ‘B’.

In the subunits, only the pseudoatoms ‘VX’ interact with the membrane beads; there is no interaction between membrane beads and ‘A’ or ‘B’ pseudoatoms. The interaction between subunit excluders and membrane beads corresponds to the repulsive part of the Lennard-Jones potential,

$$\begin{array}{c} U_{\text{g-m}}^{\text{ex}}\left(r\right)=\sum_{i}\sum_{j}4\epsilon_{\text{ex}}\left[{\left(\frac{b_{i,j}^{\text{g-m}}}{r}\right)}^{12}-{\left(\frac{b_{i,j}^{\text{g-m}}}{r}\right)}^{6}\right], \end{array}$$ (14)

where *i* runs over all lipid beads and *j* over all ‘VX’ pseudoatoms, and $b_{i,j}^{\text{g-m}}=0.5+r_{\text{in}}$ for the inner excluders VX_in_ and *b*_*i*,*j*_ = 0.5 + *r*_in_ for the outer excluders VX_out_.

### Modulus values

The mean curvature modulus for this model was calculated in Ref. [1] to be *κ* ≈ 25.66*ϵ*_gg_, and the calculation in Ref. [39] for a related model shows that $\bar{\kappa }\approx -\kappa$. However, there is an additional energetic penalty (not present in the Helfrich hamiltonian) for regions in which the two principle curvature are mismatched, so the continuum description of the bending energy goes as [39].

$$\begin{array}{c} U_{\text{bend}}/A=\kappa [\frac{1}{2}{\left(2H-2/R_{0}\right)}^{2}+\left(H^{2}-K\right)]. \end{array}$$ (15)

### Solution of the continuity equation

For a catenoid surface of revolution, the determinant *g* of the metric tensor obeys $\sqrt{g(s)}=\sqrt{c{\left(\alpha \right)}^{2}+s^{2}}$ with *c*(*α*) = *R*_0_ sin^2^(*α*) the minimum radius of the neck. The current conservation equation then reduces to

$$\begin{array}{c} \phi^{\prime }\left(s\right)+\phi \left(s\right)\beta U^{\prime }\left(s\right)=\frac{I(\alpha )/2\pi D}{\sqrt{c{\left(\alpha \right)}^{2}+s^{2}}} \end{array}$$ (16)

where *I*(*α*) is the total incoming current that we need to determine. This equation is solved by the Ansatz *ϕ*(*s*) = *p*(*s*)*e*^−*βU*(*s*)^ where $p^{\prime }\left(s\right)=\left(I\left(\alpha \right)/2\pi D\right)\frac{e^{\beta U(s)}}{\sqrt{s^{2}+c{\left(\alpha \right)}^{2}}}$. Impose absorber boundary conditions *ϕ*(*s*_*m*_) = 0 along the growth interface at *s* = *s*_*m*_ and set *ϕ*(*s*_*M*_) = *ϕ*_0_ far outside the neck area at *s* = *s*_*M*_, where we also place the zero of the potential energy (so *U*(*s*_*M*_) = 0). This gives for the current:

$$\begin{array}{c} I\left(\alpha \right)/I_{0}=\frac{1}{\int_{s_{m}}^{s_{M}}\frac{e^{\beta U(s)}}{\sqrt{s^{2}+c{\left(\alpha \right)}^{2}}}\;ds} \end{array}$$ (17)

with *I*_0_ = 2*πDϕ*_0_. If the metric factor $\sqrt{s^{2}+c{\left(\alpha \right)}^{2}}$ is set to one in Eq 17, then it reduces to a form similar to the Kramers expression for steady-state diffusion in a potential [64]. The non-entropic contribution to the chemical potential of a protein is given by $U=\frac{\delta E([\phi )]}{\delta \phi }$ where *E*([*ϕ*)] is the internal energy of the lipid bilayer outside the capsid but with a low concentration of proteins. By repeating the symmetry arguments that are used to obtain the Helfrich bending energy for a curved membrane [19], one finds that *E*([*ϕ*)] must have the general form

$$\begin{array}{c} E\left(\left[\phi \right]\right)=E_{0}\left(\left[\phi \right]\right)+\int_{A}\left[2\kappa \left(\phi \right){\left(H-C\left(\phi \right)\right)}^{2}+\bar{\kappa }\left(\phi \right)K\right]dA, \end{array}$$ (18)

Here, *E*_0_([*ϕ*]) is the protein-membrane interaction energy of a flat membrane while *κ*(*ϕ*) and $\bar{\kappa }\left(\phi \right)$ are the concentration-dependent curvature moduli. Finally, *C*(*ϕ*) is the concentration-dependent spontaneous curvature. In the limit *ϕ* = 0, all these quantities should reduce to the values appropriate for a pure lipid bilayer with capsid proteins. Expanding to lowest order in *ϕ* around this state gives

$$\begin{array}{c} \begin{array}{cc} & E_{0}\left(\left[\phi \right]\right)=E^{\prime }\int_{A}\phi dA+...\\ & \kappa \left(\phi \right)=\kappa_{L}+\kappa^{\prime }\phi +...\\ & \bar{\kappa }\left(\phi \right)={\bar{\kappa }}_{L}+\bar{\kappa^{\prime }}\phi +...\\ & C\left(\phi \right)=C^{\prime }\phi +... \end{array} \end{array}$$ (19)

It follows that

$$\begin{array}{c} \begin{array}{cc} U(\phi \to 0) & =\frac{\delta E([\phi =0])}{\delta \phi }\\ & =2\kappa^{\prime }H^{2}-4\kappa_{L}C^{\prime }H+{\bar{\kappa }}^{\prime }K \end{array} \end{array}$$ (20)

In the last step we assumed that at *s* = *s*_*M*_, where *U* = 0, the membrane is flat so *H* = *K* = 0, which means that $E_{0}^{\prime }=0$. Finally, since *H* = 0 for a minimal surface, we arrive at the simple result that $U\left(s\right)={\bar{\kappa }}^{\prime }K\left(s\right)$ to first order in *ϕ*. Since we saw earlier that ${\bar{\kappa }}_{C}$ must be negative for a stable spherical cap state, we conclude that ${\bar{\kappa }}^{\prime }<0$. For a catenoid of revolution the Gaussian curvature is given by $K\left(s\right)=-\frac{c{\left(\alpha \right)}^{2}}{{\left(s^{2}+c{\left(\alpha \right)}^{2}\right)}^{2}}$ so $U\left(s\right)={\bar{\kappa }}^{\prime }K\left(s\right)$ has a maximum at the center of the neck (*s* = 0). There is thus a “geometrical” energy barrier that the diffusing proteins need to overcome before they can be absorbed at the growth interface.

For reference, consider first the case where coupling to the Gaussian curvature is neglected. In that case, Eq 17 for *U* = 0 reduces to

$$\begin{array}{c} \begin{array}{cc} I{\left(\alpha \right)}_{{\bar{\kappa }}^{\prime }=0}/I_{0} & ≃1/\ln(s_{M}/c\left(\alpha \right))\\ & ≃1/\ln(s_{M}/\left(R_{0}\left[{\left(\rho /2R_{0}\right)}^{2}\left(1-{\left(\rho /2R_{0}\right)}^{2}\right)\right]\right)) \end{array} \end{array}$$ (21)

In the last step we used the fact that *ρ*/*R*_0_ = 2 cos *α*/2. The appearance of a logarithmic dependence on the system size *s*_*M*_ is typical of two-dimensional diffusion problems. The current first increases with *ρ* until the capsid has the shape of hemisphere at the point (*ρ*/2*R*_0_)^2^ = 1/2. Afterwards, the current decreases back to zero. A plot of the current *I* as a function of *ρ*^2^ is symmetrical around the midpoint maximum $\rho^{2}=2R_{0}^{2}$.

Now consider the case that $\beta |{\bar{\kappa }}^{\prime }|/{\left(R_{0}\sin\alpha \right)}^{2}$ is comparable to, or larger than, one. Using the steepest descent method around the maximum of *U*(*s*) at *s* = 0 leads to

$$\begin{array}{c} \begin{array}{cc} I\left(\alpha \right)/I_{0} & ≃\frac{\sqrt{\beta |{\bar{\kappa }}^{\prime }}|}{c\left(\alpha \right)}e^{-\beta |{\bar{\kappa }}^{\prime }|/c{\left(\alpha \right)}^{2}}\\ & ≃\frac{\sqrt{\beta |{\bar{\kappa }}^{\prime }|}}{R_{0}{\text{sin}}^{2}\alpha }e^{-\beta |{\bar{\kappa }}^{\prime }|/{\left(R_{0}{\text{sin}}^{2}\alpha \right)}^{2}} \end{array} \end{array}$$ (22)

The current as a function of the aperture angle has an essential singularity at the pinch-off point *α* = 0 and *ρ*/*R*_0_ = 2, where it precipitously drops to zero.
